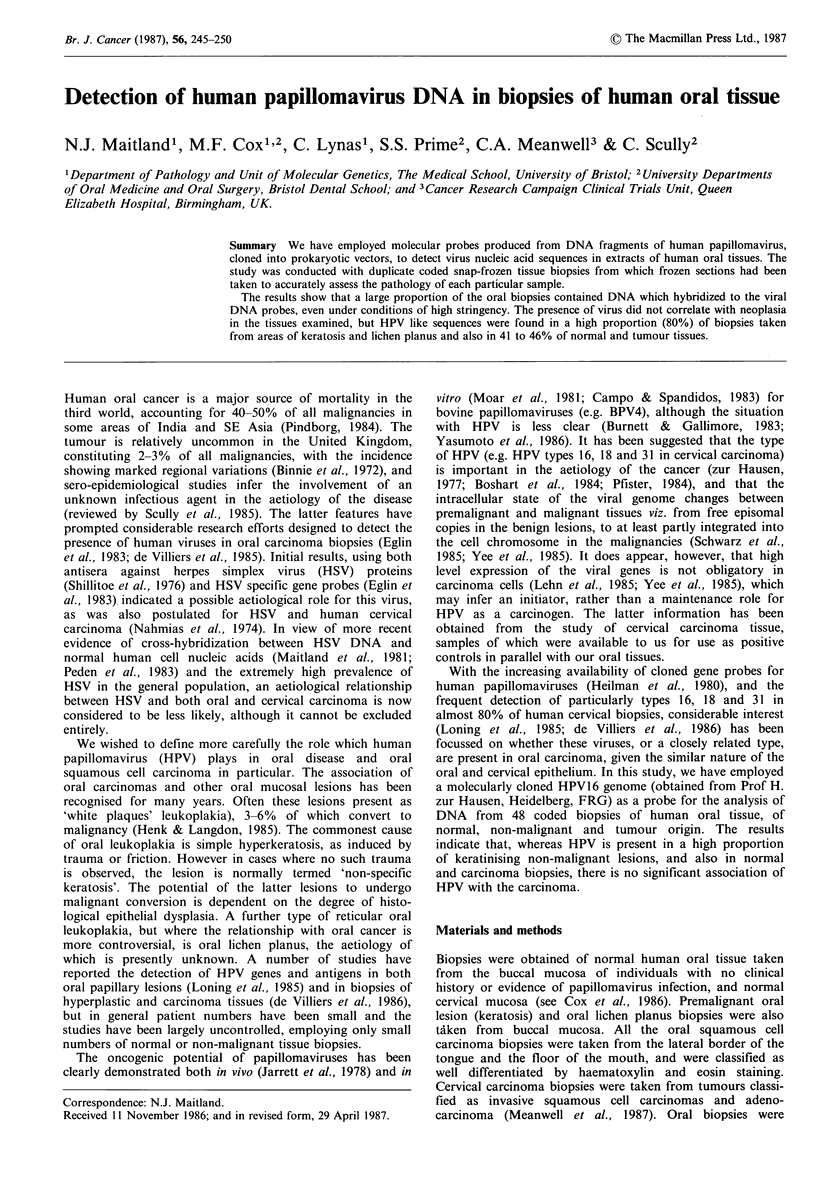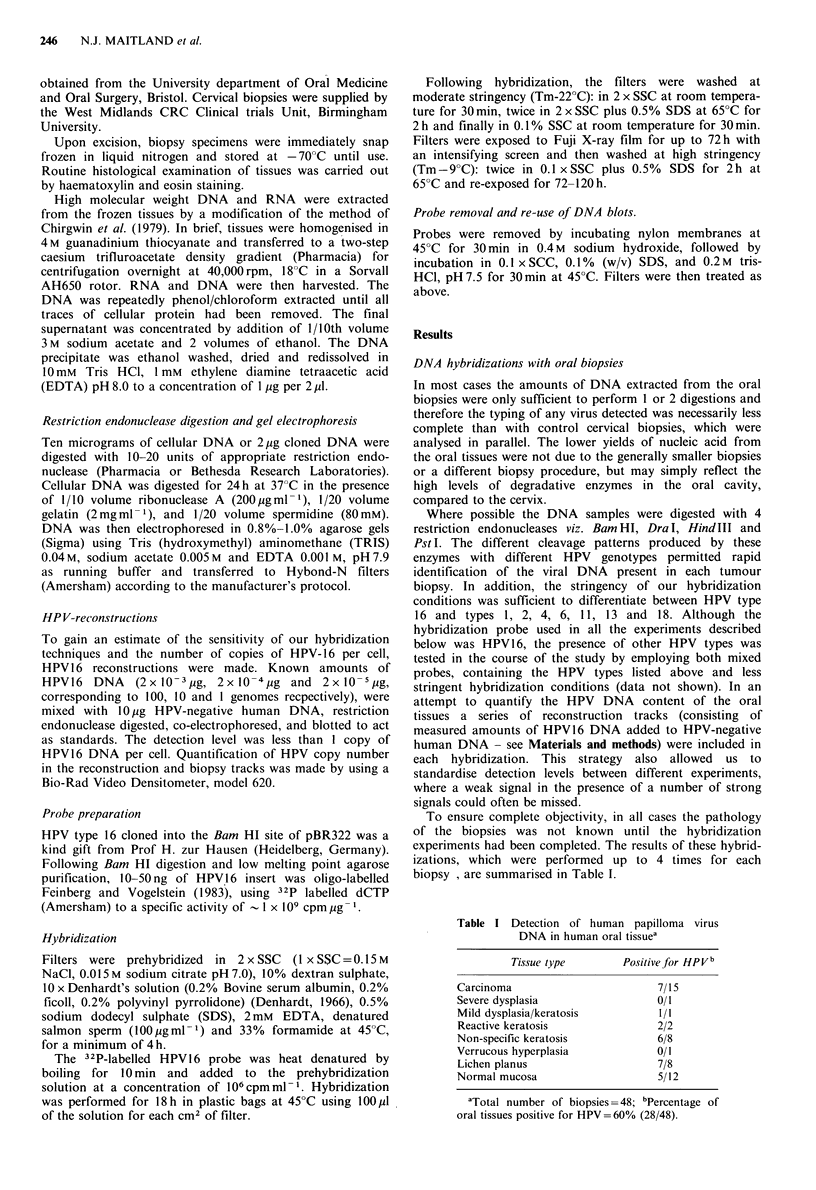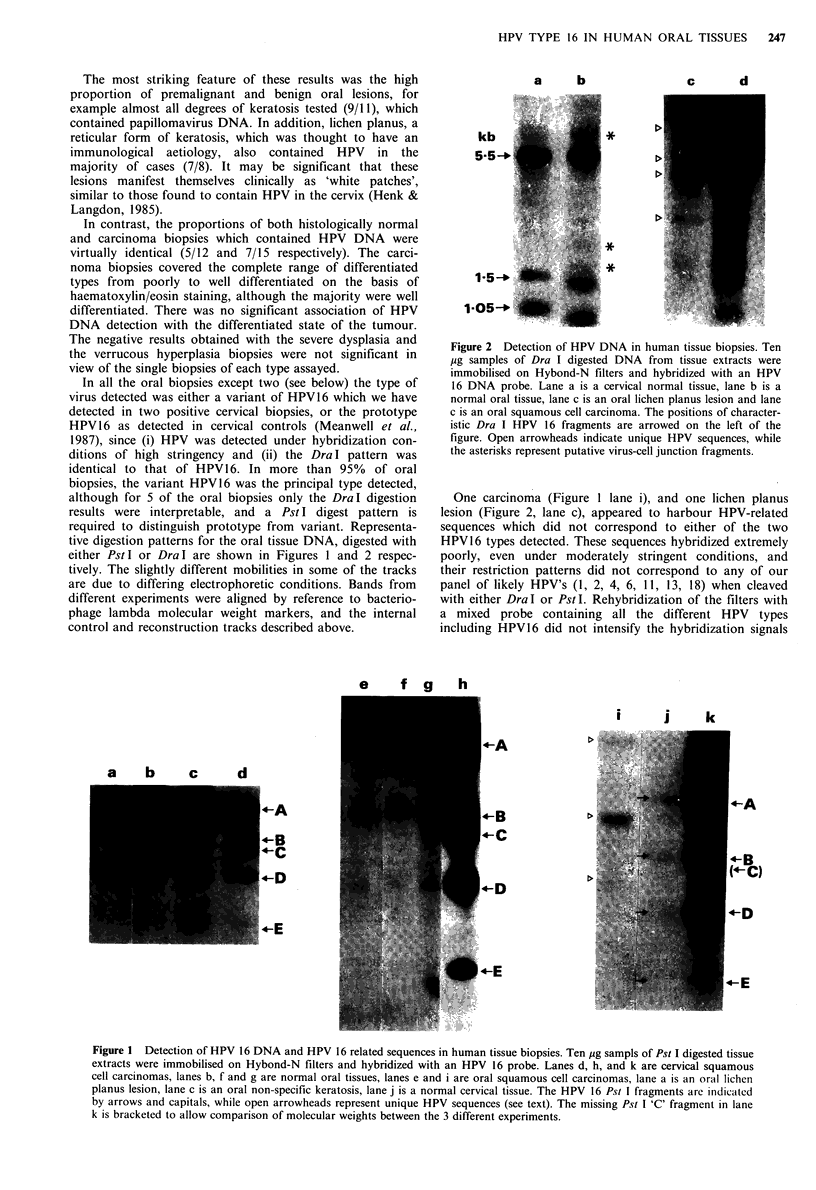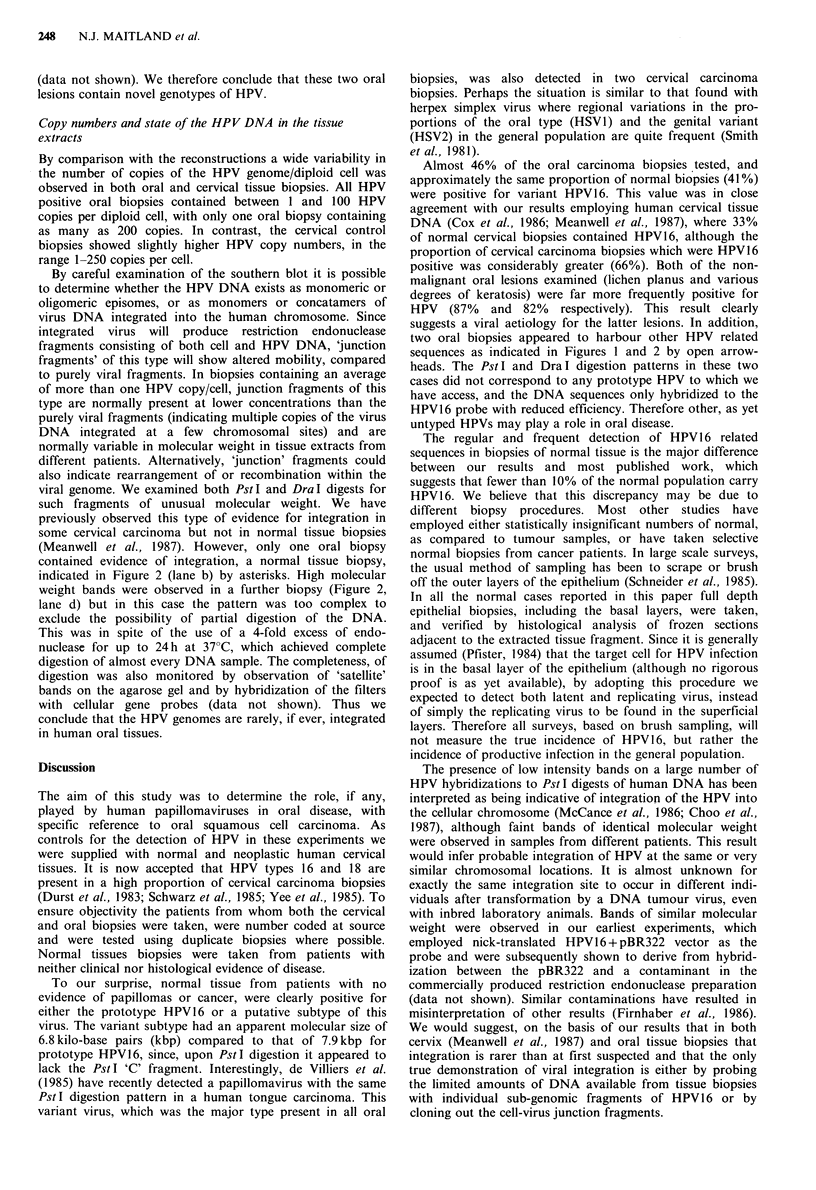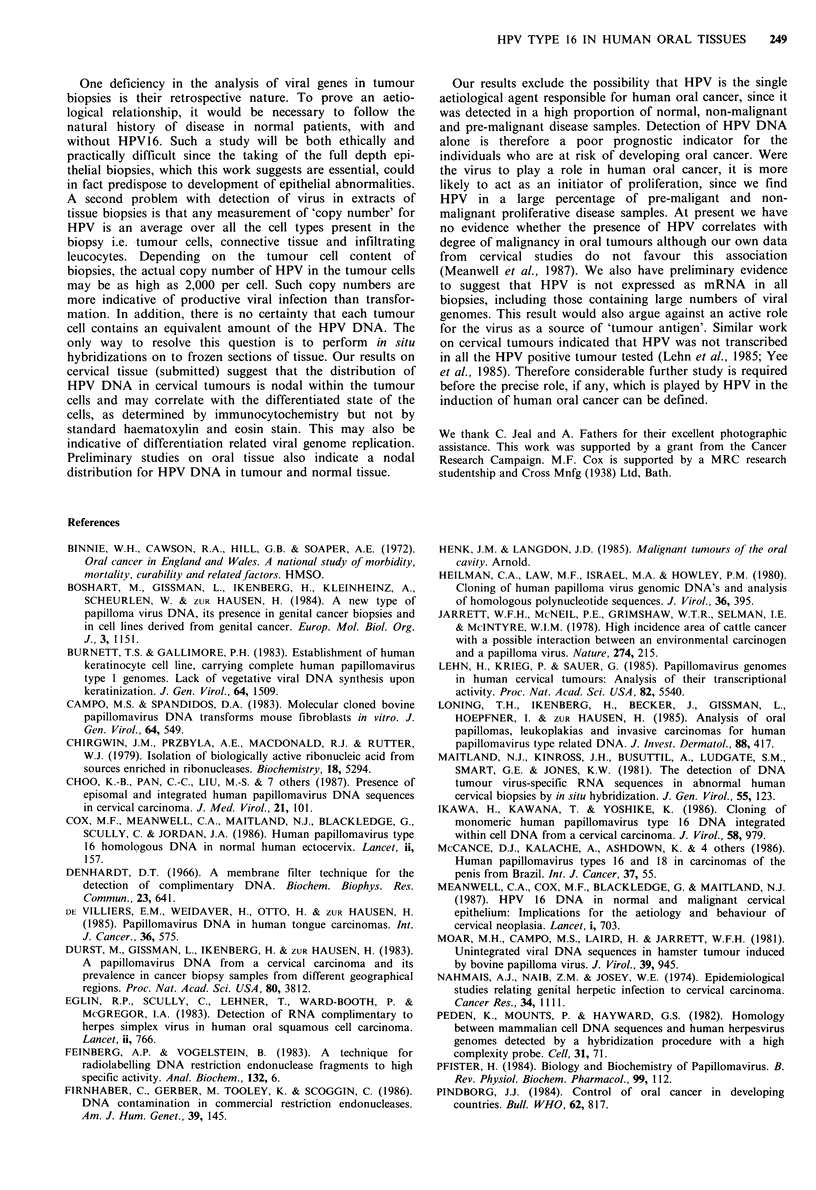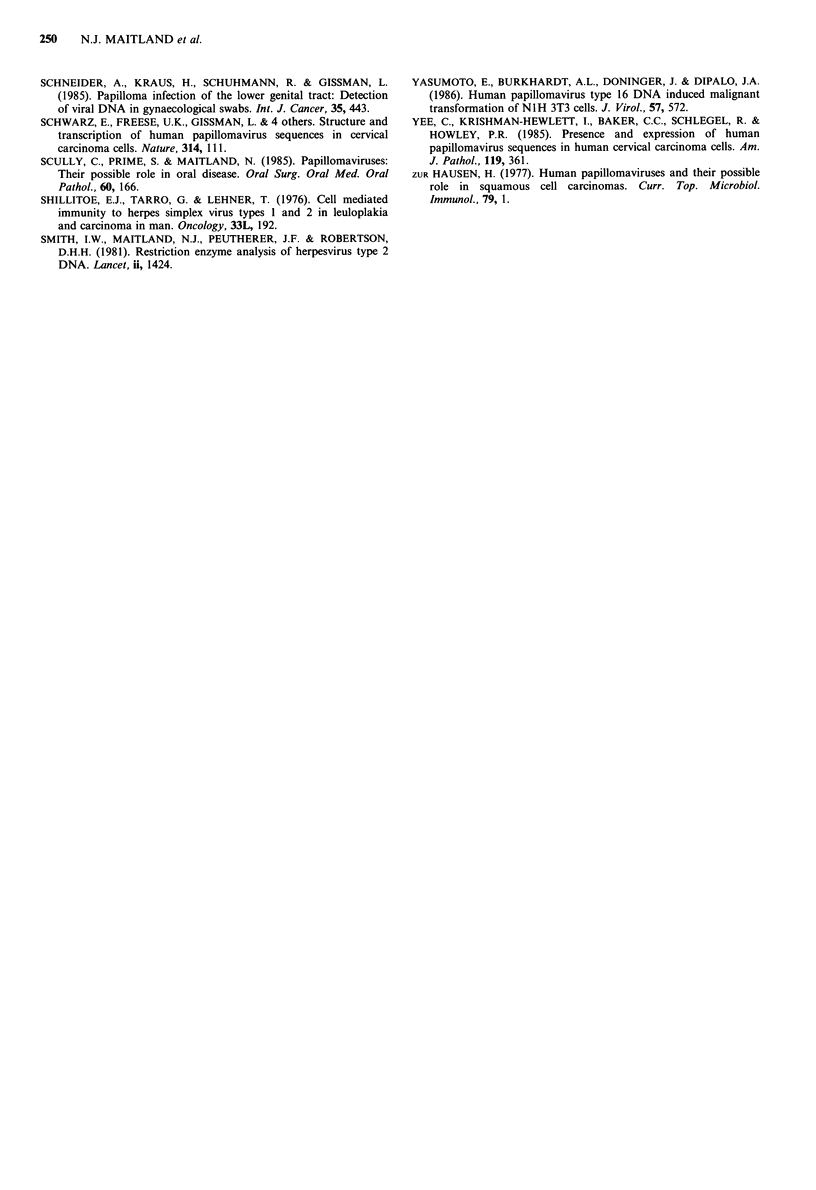# Detection of human papillomavirus DNA in biopsies of human oral tissue.

**DOI:** 10.1038/bjc.1987.185

**Published:** 1987-09

**Authors:** N. J. Maitland, M. F. Cox, C. Lynas, S. S. Prime, C. A. Meanwell, C. Scully

**Affiliations:** Department of Pathology, Medical School, University of Bristol, UK.

## Abstract

**Images:**


*Br. J. Cancer* (1987), **56**, 245–250 © The Macmillan Press Ltd., 1987

### Detection of human papillomavirus DNA in biopsies of human oral tissue

N.J. Maitland[1], M.F. Cox[1,2], C. Lynas[1], S.S. Prime[2], C.A. Meanwell[3] & C. Scully[2]

[1]*Department of Pathology and Unit of Molecular Genetics, The Medical School, University of Bristol;* [2]*University Departments of Oral Medicine and Oral Surgery, Bristol Dental School; and* [3]*Cancer Research Campaign Clinical Trials Unit, Queen Elizabeth Hospital, Birmingham, UK.*

**Summary** We have employed molecular probes produced from DNA fragments of human papillomavirus, cloned into prokaryotic vectors, to detect virus nucleic acid sequences in extracts of human oral tissues. The study was conducted with duplicate coded snap-frozen tissue biopsies from which frozen sections had been taken to accurately assess the pathology of each particular sample.

The results show that a large proportion of the oral biopsies contained DNA which hybridized to the viral DNA probes, even under conditions of high stringency. The presence of virus did not correlate with neoplasia in the tissues examined, but HPV like sequences were found in a high proportion (80%) of biopsies taken from areas of keratosis and lichen planus and also in 41 to 46% of normal and tumour tissues.

Human oral cancer is a major source of mortality in the third world, accounting for 40–50% of all malignancies in some areas of India and SE Asia (Pindborg, 1984). The tumour is relatively uncommon in the United Kingdom, constituting 2–3% of all malignancies, with the incidence showing marked regional variations (Binnie *et al.*, 1972), and sero-epidemiological studies infer the involvement of an unknown infectious agent in the aetiology of the disease (reviewed by Scully *et al.*, 1985). The latter features have prompted considerable research efforts designed to detect the presence of human viruses in oral carcinoma biopsies (Eglin *et al.*, 1983; de Villiers *et al.*, 1985). Initial results, using both antisera against herpes simplex virus (HSV) proteins (Shillitoe *et al.*, 1976) and HSV specific gene probes (Eglin *et al.*, 1983) indicated a possible aetiological role for this virus, as was also postulated for HSV and human cervical carcinoma (Nahmias *et al.*, 1974). In view of more recent evidence of cross-hybridization between HSV DNA and normal human cell nucleic acids (Maitland *et al.*, 1981; Peden *et al.*, 1983) and the extremely high prevalence of HSV in the general population, an aetiological relationship between HSV and both oral and cervical carcinoma is now considered to be less likely, although it cannot be excluded entirely.

We wished to define more carefully the role which human papillomavirus (HPV) plays in oral disease and oral squamous cell carcinoma in particular. The association of oral carcinomas and other oral mucosal lesions has been recognised for many years. Often these lesions present as 'white plaques' leukoplakia), 3–6% of which convert to malignancy (Henk & Langdon, 1985). The commonest cause of oral leukoplakia is simple hyperkeratosis, as induced by trauma or friction. However in cases where no such trauma is observed, the lesion is normally termed 'non-specific keratosis'. The potential of the latter lesions to undergo malignant conversion is dependent on the degree of histological epithelial dysplasia. A further type of reticular oral leukoplakia, but where the relationship with oral cancer is more controversial, is oral lichen planus, the aetiology of which is presently unknown. A number of studies have reported the detection of HPV genes and antigens in both oral papillary lesions (Loning *et al.*, 1985) and in biopsies of hyperplastic and carcinoma tissues (de Villiers *et al.*, 1986), but in general patient numbers have been small and the studies have been largely uncontrolled, employing only small numbers of normal or non-malignant tissue biopsies.

The oncogenic potential of papillomaviruses has been clearly demonstrated both *in vivo* (Jarrett *et al.*, 1978) and *in vitro* (Moar *et al.*, 1981; Campo & Spandidos, 1983) for bovine papillomaviruses (e.g. BPV4), although the situation with HPV is less clear (Burnett & Gallimore, 1983; Yasumoto *et al.*, 1986). It has been suggested that the type of HPV (e.g. HPV types 16, 18 and 31 in cervical carcinoma) is important in the aetiology of the cancer (zur Hausen, 1977; Boshart *et al.*, 1984; Pfister, 1984), and that the intracellular state of the viral genome changes between premalignant and malignant tissues *viz.* from free episomal copies in the benign lesions, to at least partly integrated into the cell chromosome in the malignancies (Schwarz *et al.*, 1985; Yee *et al.*, 1985). It does appear, however, that high level expression of the viral genes is not obligatory in carcinoma cells (Lehn *et al.*, 1985; Yee *et al.*, 1985), which may infer an initiator, rather than a maintenance role for HPV as a carcinogen. The latter information has been obtained from the study of cervical carcinoma tissue, samples of which were available to us for use as positive controls in parallel with our oral tissues.

With the increasing availability of cloned gene probes for human papillomaviruses (Heilman *et al.*, 1980), and the frequent detection of particularly types 16, 18 and 31 in almost 80% of human cervical biopsies, considerable interest (Loning *et al.*, 1985; de Villiers *et al.*, 1986) has been focussed on whether these viruses, or a closely related type, are present in oral carcinoma, given the similar nature of the oral and cervical epithelium. In this study, we have employed a molecularly cloned HPV16 genome (obtained from Prof H. zur Hausen, Heidelberg, FRG) as a probe for the analysis of DNA from 48 coded biopsies of human oral tissue, of normal, non-malignant and tumour origin. The results indicate that, whereas HPV is present in a high proportion of keratinising non-malignant lesions, and also in normal and carcinoma biopsies, there is no significant association of HPV with the carcinoma.

### Materials and methods

Biopsies were obtained of normal human oral tissue taken from the buccal mucosa of individuals with no clinical history or evidence of papillomavirus infection, and normal cervical mucosa (see Cox *et al.*, 1986). Premalignant oral lesion (keratosis) and oral lichen planus biopsies were also tåken from buccal mucosa. All the oral squamous cell carcinoma biopsies were taken from the lateral border of the tongue and the floor of the mouth, and were classified as well differentiated by haematoxylin and eosin staining. Cervical carcinoma biopsies were taken from tumours classified as invasive squamous cell carcinomas and adenocarcinoma (Meanwell *et al.*, 1987). Oral biopsies were

Correspondence: N.J. Maitland.
Received 11 November 1986; and in revised form, 29 April 1987.

obtained from the University department of Oral Medicine and Oral Surgery, Bristol. Cervical biopsies were supplied by the West Midlands CRC Clinical trials Unit, Birmingham University.

Upon excision, biopsy specimens were immediately snap frozen in liquid nitrogen and stored at −70°C until use. Routine histological examination of tissues was carried out by haematoxylin and eosin staining.

High molecular weight DNA and RNA were extracted from the frozen tissues by a modification of the method of Chirgwin *et al.* (1979). In brief, tissues were homogenised in 4 M guanadinium thiocyanate and transferred to a two-step caesium trifluroacetate density gradient (Pharmacia) for centrifugation overnight at 40,000 rpm, 18°C in a Sorvall AH650 rotor. RNA and DNA were then harvested. The DNA was repeatedly phenol/chloroform extracted until all traces of cellular protein had been removed. The final supernatant was concentrated by addition of 1/10th volume 3 M sodium acetate and 2 volumes of ethanol. The DNA precipitate was ethanol washed, dried and redissolved in 10 mM Tris HCl, 1 mM ethylene diamine tetraacetic acid (EDTA) pH 8.0 to a concentration of 1 $\mu$g per 2 $\mu$l.

*Restriction endonuclease digestion and gel electrophoresis*

Ten micrograms of cellular DNA or 2 $\mu$g cloned DNA were digested with 10–20 units of appropriate restriction endonuclease (Pharmacia or Bethesda Research Laboratories). Cellular DNA was digested for 24 h at 37°C in the presence of 1/10 volume ribonuclease A (200 $\mu g\,ml^{-1}$), 1/20 volume gelatin (2 $mg\,ml^{-1}$), and 1/20 volume spermidine (80 mM). DNA was then electrophoresed in 0.8%–1.0% agarose gels (Sigma) using Tris (hydroxymethyl) aminomethane (TRIS) 0.04 M, sodium acetate 0.005 M and EDTA 0.001 M, pH 7.9 as running buffer and transferred to Hybond-N filters (Amersham) according to the manufacturer's protocol.

*HPV-reconstructions*

To gain an estimate of the sensitivity of our hybridization techniques and the number of copies of HPV-16 per cell, HPV16 reconstructions were made. Known amounts of HPV16 DNA ($2\times10^{-3}\,\mu g$, $2\times10^{-4}\,\mu g$ and $2\times10^{-5}\,\mu g$, corresponding to 100, 10 and 1 genomes recpectively), were mixed with 10 $\mu$g HPV-negative human DNA, restriction endonuclease digested, co-electrophoresed, and blotted to act as standards. The detection level was less than 1 copy of HPV16 DNA per cell. Quantification of HPV copy number in the reconstruction and biopsy tracks was made by using a Bio-Rad Video Densitometer, model 620.

*Probe preparation*

HPV type 16 cloned into the *Bam* HI site of pBR322 was a kind gift from Prof H. zur Hausen (Heidelberg, Germany). Following *Bam* HI digestion and low melting point agarose purification, 10–50 ng of HPV16 insert was oligo-labelled Feinberg and Vogelstein (1983), using $^{32}P$ labelled dCTP (Amersham) to a specific activity of $\sim 1\times10^{9}$ cpm $\mu g^{-1}$.

*Hybridization*

Filters were prehybridized in 2 × SSC (1 × SSC = 0.15 M NaCl, 0.015 M sodium citrate pH 7.0), 10% dextran sulphate, 10 × Denhardt's solution (0.2% Bovine serum albumin, 0.2% ficoll, 0.2% polyvinyl pyrrolidone) (Denhardt, 1966), 0.5% sodium dodecyl sulphate (SDS), 2 mM EDTA, denatured salmon sperm (100 $\mu g\,ml^{-1}$) and 33% formamide at 45°C, for a minimum of 4 h.

The $^{32}P$-labelled HPV16 probe was heat denatured by boiling for 10 min and added to the prehybridization solution at a concentration of $10^{6}$ cpm $ml^{-1}$. Hybridization was performed for 18 h in plastic bags at 45°C using 100 $\mu$l of the solution for each $cm^2$ of filter.

Following hybridization, the filters were washed at moderate stringency (Tm-22°C): in 2 × SSC at room temperature for 30 min, twice in 2 × SSC plus 0.5% SDS at 65°C for 2 h and finally in 0.1% SSC at room temperature for 30 min. Filters were exposed to Fuji X-ray film for up to 72 h with an intensifying screen and then washed at high stringency (Tm − 9°C): twice in 0.1 × SSC plus 0.5% SDS for 2 h at 65°C and re-exposed for 72–120 h.

*Probe removal and re-use of DNA blots.*

Probes were removed by incubating nylon membranes at 45°C for 30 min in 0.4 M sodium hydroxide, followed by incubation in 0.1 × SCC, 0.1% (w/v) SDS, and 0.2 M tris-HCl, pH 7.5 for 30 min at 45°C. Filters were then treated as above.

### Results

*DNA hybridizations with oral biopsies*

In most cases the amounts of DNA extracted from the oral biopsies were only sufficient to perform 1 or 2 digestions and therefore the typing of any virus detected was necessarily less complete than with control cervical biopsies, which were analysed in parallel. The lower yields of nucleic acid from the oral tissues were not due to the generally smaller biopsies or a different biopsy procedure, but may simply reflect the high levels of degradative enzymes in the oral cavity, compared to the cervix.

Where possible the DNA samples were digested with 4 restriction endonucleases *viz. Bam*HI, *Dra*I, *Hind*III and *Pst*I. The different cleavage patterns produced by these enzymes with different HPV genotypes permitted rapid identification of the viral DNA present in each tumour biopsy. In addition, the stringency of our hybridization conditions was sufficient to differentiate between HPV type 16 and types 1, 2, 4, 6, 11, 13 and 18. Although the hybridization probe used in all the experiments described below was HPV16, the presence of other HPV types was tested in the course of the study by employing both mixed probes, containing the HPV types listed above and less stringent hybridization conditions (data not shown). In an attempt to quantify the HPV DNA content of the oral tissues a series of reconstruction tracks (consisting of measured amounts of HPV16 DNA added to HPV-negative human DNA – see **Materials and methods**) were included in each hybridization. This strategy also allowed us to standardise detection levels between different experiments, where a weak signal in the presence of a number of strong signals could often be missed.

To ensure complete objectivity, in all cases the pathology of the biopsies was not known until the hybridization experiments had been completed. The results of these hybridizations, which were performed up to 4 times for each biopsy , are summarised in Table I.

**Table I** Detection of human papilloma virus DNA in human oral tissue[a]

| *Tissue type* | *Positive for HPV*[b] |
|---|---|
| Carcinoma | 7/15 |
| Severe dysplasia | 0/1 |
| Mild dysplasia/keratosis | 1/1 |
| Reactive keratosis | 2/2 |
| Non-specific keratosis | 6/8 |
| Verrucous hyperplasia | 0/1 |
| Lichen planus | 7/8 |
| Normal mucosa | 5/12 |

[a]Total number of biopsies = 48; [b]Percentage of oral tissues positive for HPV = 60% (28/48).

The most striking feature of these results was the high proportion of premalignant and benign oral lesions, for example almost all degrees of keratosis tested (9/11), which contained papillomavirus DNA. In addition, lichen planus, a reticular form of keratosis, which was thought to have an immunological aetiology, also contained HPV in the majority of cases (7/8). It may be significant that these lesions manifest themselves clinically as 'white patches', similar to those found to contain HPV in the cervix (Henk & Langdon, 1985).

In contrast, the proportions of both histologically normal and carcinoma biopsies which contained HPV DNA were virtually identical (5/12 and 7/15 respectively). The carcinoma biopsies covered the complete range of differentiated types from poorly to well differentiated on the basis of haematoxylin/eosin staining, although the majority were well differentiated. There was no significant association of HPV DNA detection with the differentiated state of the tumour. The negative results obtained with the severe dysplasia and the verrucous hyperplasia biopsies were not significant in view of the single biopsies of each type assayed.

In all the oral biopsies except two (see below) the type of virus detected was either a variant of HPV16 which we have detected in two positive cervical biopsies, or the prototype HPV16 as detected in cervical controls (Meanwell *et al.*, 1987), since (i) HPV was detected under hybridization conditions of high stringency and (ii) the *Dra* I pattern was identical to that of HPV16. In more than 95% of oral biopsies, the variant HPV16 was the principal type detected, although for 5 of the oral biopsies only the *Dra* I digestion results were interpretable, and a *Pst* I digest pattern is required to distinguish prototype from variant. Representative digestion patterns for the oral tissue DNA, digested with either *Pst* I or *Dra* I are shown in Figures 1 and 2 respectively. The slightly different mobilities in some of the tracks are due to differing electrophoretic conditions. Bands from different experiments were aligned by reference to bacteriophage lambda molecular weight markers, and the internal control and reconstruction tracks described above.

**Figure 2** Detection of HPV DNA in human tissue biopsies. Ten μg samples of *Dra* I digested DNA from tissue extracts were immobilised on Hybond-N filters and hybridized with an HPV 16 DNA probe. Lane a is a cervical normal tissue, lane b is a normal oral tissue, lane c is an oral lichen planus lesion and lane c is an oral squamous cell carcinoma. The positions of characteristic *Dra* I HPV 16 fragments are arrowed on the left of the figure. Open arrowheads indicate unique HPV sequences, while the asterisks represent putative virus-cell junction fragments.

One carcinoma (Figure 1 lane i), and one lichen planus lesion (Figure 2, lane c), appeared to harbour HPV-related sequences which did not correspond to either of the two HPV16 types detected. These sequences hybridized extremely poorly, even under moderately stringent conditions, and their restriction patterns did not correspond to any of our panel of likely HPV's (1, 2, 4, 6, 11, 13, 18) when cleaved with either *Dra* I or *Pst* I. Rehybridization of the filters with a mixed probe containing all the different HPV types including HPV16 did not intensify the hybridization signals

**Figure 1** Detection of HPV 16 DNA and HPV 16 related sequences in human tissue biopsies. Ten μg sampls of *Pst* I digested tissue extracts were immobilised on Hybond-N filters and hybridized with an HPV 16 probe. Lanes d, h, and k are cervical squamous cell carcinomas, lanes b, f and g are normal oral tissues, lanes e and i are oral squamous cell carcinomas, lane a is an oral lichen planus lesion, lane c is an oral non-specific keratosis, lane j is a normal cervical tissue. The HPV 16 *Pst* I fragments are indicated by arrows and capitals, while open arrowheads represent unique HPV sequences (see text). The missing *Pst* I 'C' fragment in lane k is bracketed to allow comparison of molecular weights between the 3 different experiments.

(data not shown). We therefore conclude that these two oral lesions contain novel genotypes of HPV.

*Copy numbers and state of the HPV DNA in the tissue extracts*

By comparison with the reconstructions a wide variability in the number of copies of the HPV genome/diploid cell was observed in both oral and cervical tissue biopsies. All HPV positive oral biopsies contained between 1 and 100 HPV copies per diploid cell, with only one oral biopsy containing as many as 200 copies. In contrast, the cervical control biopsies showed slightly higher HPV copy numbers, in the range 1–250 copies per cell.

By careful examination of the southern blot it is possible to determine whether the HPV DNA exists as monomeric or oligomeric episomes, or as monomers or concatamers of virus DNA integrated into the human chromosome. Since integrated virus will produce restriction endonuclease fragments consisting of both cell and HPV DNA, 'junction fragments' of this type will show altered mobility, compared to purely viral fragments. In biopsies containing an average of more than one HPV copy/cell, junction fragments of this type are normally present at lower concentrations than the purely viral fragments (indicating multiple copies of the virus DNA integrated at a few chromosomal sites) and are normally variable in molecular weight in tissue extracts from different patients. Alternatively, 'junction' fragments could also indicate rearrangement of or recombination within the viral genome. We examined both *Pst* I and *Dra* I digests for such fragments of unusual molecular weight. We have previously observed this type of evidence for integration in some cervical carcinoma but not in normal tissue biopsies (Meanwell *et al.*, 1987). However, only one oral biopsy contained evidence of integration, a normal tissue biopsy, indicated in Figure 2 (lane b) by asterisks. High molecular weight bands were observed in a further biopsy (Figure 2, lane d) but in this case the pattern was too complex to exclude the possibility of partial digestion of the DNA. This was in spite of the use of a 4-fold excess of endonuclease for up to 24 h at 37°C, which achieved complete digestion of almost every DNA sample. The completeness, of digestion was also monitored by observation of 'satellite' bands on the agarose gel and by hybridization of the filters with cellular gene probes (data not shown). Thus we conclude that the HPV genomes are rarely, if ever, integrated in human oral tissues.

### Discussion

The aim of this study was to determine the role, if any, played by human papillomaviruses in oral disease, with specific reference to oral squamous cell carcinoma. As controls for the detection of HPV in these experiments we were supplied with normal and neoplastic human cervical tissues. It is now accepted that HPV types 16 and 18 are present in a high proportion of cervical carcinoma biopsies (Durst *et al.*, 1983; Schwarz *et al.*, 1985; Yee *et al.*, 1985). To ensure objectivity the patients from whom both the cervical and oral biopsies were taken, were number coded at source and were tested using duplicate biopsies where possible. Normal tissues biopsies were taken from patients with neither clinical nor histological evidence of disease.

To our surprise, normal tissue from patients with no evidence of papillomas or cancer, were clearly positive for either the prototype HPV16 or a putative subtype of this virus. The variant subtype had an apparent molecular size of 6.8 kilo-base pairs (kbp) compared to that of 7.9 kbp for prototype HPV16, since, upon *Pst* I digestion it appeared to lack the *Pst* I 'C' fragment. Interestingly, de Villiers *et al.* (1985) have recently detected a papillomavirus with the same *Pst* I digestion pattern in a human tongue carcinoma. This variant virus, which was the major type present in all oral biopsies, was also detected in two cervical carcinoma biopsies. Perhaps the situation is similar to that found with herpex simplex virus where regional variations in the proportions of the oral type (HSV1) and the genital variant (HSV2) in the general population are quite frequent (Smith *et al.*, 1981).

Almost 46% of the oral carcinoma biopsies tested, and approximately the same proportion of normal biopsies (41%) were positive for variant HPV16. This value was in close agreement with our results employing human cervical tissue DNA (Cox *et al.*, 1986; Meanwell *et al.*, 1987), where 33% of normal cervical biopsies contained HPV16, although the proportion of cervical carcinoma biopsies which were HPV16 positive was considerably greater (66%). Both of the non-malignant oral lesions examined (lichen planus and various degrees of keratosis) were far more frequently positive for HPV (87% and 82% respectively). This result clearly suggests a viral aetiology for the latter lesions. In addition, two oral biopsies appeared to harbour other HPV related sequences as indicated in Figures 1 and 2 by open arrowheads. The *Pst* I and Dra I digestion patterns in these two cases did not correspond to any prototype HPV to which we have access, and the DNA sequences only hybridized to the HPV16 probe with reduced efficiency. Therefore other, as yet untyped HPVs may play a role in oral disease.

The regular and frequent detection of HPV16 related sequences in biopsies of normal tissue is the major difference between our results and most published work, which suggests that fewer than 10% of the normal population carry HPV16. We believe that this discrepancy may be due to different biopsy procedures. Most other studies have employed either statistically insignificant numbers of normal, as compared to tumour samples, or have taken selective normal biopsies from cancer patients. In large scale surveys, the usual method of sampling has been to scrape or brush off the outer layers of the epithelium (Schneider *et al.*, 1985). In all the normal cases reported in this paper full depth epithelial biopsies, including the basal layers, were taken, and verified by histological analysis of frozen sections adjacent to the extracted tissue fragment. Since it is generally assumed (Pfister, 1984) that the target cell for HPV infection is in the basal layer of the epithelium (although no rigorous proof is as yet available), by adopting this procedure we expected to detect both latent and replicating virus, instead of simply the replicating virus to be found in the superficial layers. Therefore all surveys, based on brush sampling, will not measure the true incidence of HPV16, but rather the incidence of productive infection in the general population.

The presence of low intensity bands on a large number of HPV hybridizations to *Pst* I digests of human DNA has been interpreted as being indicative of integration of the HPV into the cellular chromosome (McCance *et al.*, 1986; Choo *et al.*, 1987), although faint bands of identical molecular weight were observed in samples from different patients. This result would infer probable integration of HPV at the same or very similar chromosomal locations. It is almost unknown for exactly the same integration site to occur in different individuals after transformation by a DNA tumour virus, even with inbred laboratory animals. Bands of similar molecular weight were observed in our earliest experiments, which employed nick-translated HPV16+pBR322 vector as the probe and were subsequently shown to derive from hybridization between the pBR322 and a contaminant in the commercially produced restriction endonuclease preparation (data not shown). Similar contaminations have resulted in misinterpretation of other results (Firnhaber *et al.*, 1986). We would suggest, on the basis of our results that in both cervix (Meanwell *et al.*, 1987) and oral tissue biopsies that integration is rarer than at first suspected and that the only true demonstration of viral integration is either by probing the limited amounts of DNA available from tissue biopsies with individual sub-genomic fragments of HPV16 or by cloning out the cell-virus junction fragments.

One deficiency in the analysis of viral genes in tumour biopsies is their retrospective nature. To prove an aetiological relationship, it would be necessary to follow the natural history of disease in normal patients, with and without HPV16. Such a study will be both ethically and practically difficult since the taking of the full depth epithelial biopsies, which this work suggests are essential, could in fact predispose to development of epithelial abnormalities. A second problem with detection of virus in extracts of tissue biopsies is that any measurement of 'copy number' for HPV is an average over all the cell types present in the biopsy i.e. tumour cells, connective tissue and infiltrating leucocytes. Depending on the tumour cell content of biopsies, the actual copy number of HPV in the tumour cells may be as high as 2,000 per cell. Such copy numbers are more indicative of productive viral infection than transformation. In addition, there is no certainty that each tumour cell contains an equivalent amount of the HPV DNA. The only way to resolve this question is to perform *in situ* hybridizations on to frozen sections of tissue. Our results on cervical tissue (submitted) suggest that the distribution of HPV DNA in cervical tumours is nodal within the tumour cells and may correlate with the differentiated state of the cells, as determined by immunocytochemistry but not by standard haematoxylin and eosin stain. This may also be indicative of differentiation related viral genome replication. Preliminary studies on oral tissue also indicate a nodal distribution for HPV DNA in tumour and normal tissue.

Our results exclude the possibility that HPV is the single aetiological agent responsible for human oral cancer, since it was detected in a high proportion of normal, non-malignant and pre-malignant disease samples. Detection of HPV DNA alone is therefore a poor prognostic indicator for the individuals who are at risk of developing oral cancer. Were the virus to play a role in human oral cancer, it is more likely to act as an initiator of proliferation, since we find HPV in a large percentage of pre-maligant and non-malignant proliferative disease samples. At present we have no evidence whether the presence of HPV correlates with degree of malignancy in oral tumours although our own data from cervical studies do not favour this association (Meanwell *et al.*, 1987). We also have preliminary evidence to suggest that HPV is not expressed as mRNA in all biopsies, including those containing large numbers of viral genomes. This result would also argue against an active role for the virus as a source of 'tumour antigen'. Similar work on cervical tumours indicated that HPV was not transcribed in all the HPV positive tumour tested (Lehn *et al.*, 1985; Yee *et al.*, 1985). Therefore considerable further study is required before the precise role, if any, which is played by HPV in the induction of human oral cancer can be defined.

We thank C. Jeal and A. Fathers for their excellent photographic assistance. This work was supported by a grant from the Cancer Research Campaign. M.F. Cox is supported by a MRC research studentship and Cross Mnfg (1938) Ltd, Bath.

### References

BINNIE, W.H., CAWSON, R.A., HILL, G.B. & SOAPER, A.E. (1972). *Oral cancer in England and Wales. A national study of morbidity, mortality, curability and related factors.* HMSO.

BOSHART, M., GISSMAN, L., IKENBERG, H., KLEINHEINZ, A., SCHEURLEN, W. & ZUR HAUSEN, H. (1984). A new type of papilloma virus DNA, its presence in genital cancer biopsies and in cell lines derived from genital cancer. *Europ. Mol. Biol. Org. J.*, **3**, 1151.

BURNETT, T.S. & GALLIMORE, P.H. (1983). Establishment of human keratinocyte cell line, carrying complete human papillomavirus type 1 genomes. Lack of vegetative viral DNA synthesis upon keratinization. *J. Gen. Virol.*, **64**, 1509.

CAMPO, M.S. & SPANDIDOS, D.A. (1983). Molecular cloned bovine papillomavirus DNA transforms mouse fibroblasts *in vitro*. *J. Gen. Virol.*, **64**, 549.

CHIRGWIN, J.M., PRZBYLA, A.E., MACDONALD, R.J. & RUTTER, W.J. (1979). Isolation of biologically active ribonucleic acid from sources enriched in ribonucleases. *Biochemistry*, **18**, 5294.

CHOO, K.-B., PAN, C.-C., LIU, M.-S. & 7 others (1987). Presence of episomal and integrated human papillomavirus DNA sequences in cervical carcinoma. *J. Med. Virol.*, **21**, 101.

COX, M.F., MEANWELL, C.A., MAITLAND, N.J., BLACKLEDGE, G., SCULLY, C. & JORDAN, J.A. (1986). Human papillomavirus type 16 homologous DNA in normal human ectocervix. *Lancet*, **ii**, 157.

DENHARDT, D.T. (1966). A membrane filter technique for the detection of complimentary DNA. *Biochem. Biophys. Res. Commun.*, **23**, 641.

DE VILLIERS, E.M., WEIDAVER, H., OTTO, H. & ZUR HAUSEN, H. (1985). Papillomavirus DNA in human tongue carcinomas. *Int. J. Cancer.*, **36**, 575.

DURST, M., GISSMAN, L., IKENBERG, H. & ZUR HAUSEN, H. (1983). A papillomavirus DNA from a cervical carcinoma and its prevalence in cancer biopsy samples from different geographical regions. *Proc. Nat. Acad. Sci. USA*, **80**, 3812.

EGLIN, R.P., SCULLY, C., LEHNER, T., WARD-BOOTH, P. & MCGREGOR, I.A. (1983). Detection of RNA complimentary to herpes simplex virus in human oral squamous cell carcinoma. *Lancet*, **ii**, 766.

FEINBERG, A.P. & VOGELSTEIN, B. (1983). A technique for radiolabelling DNA restriction endonuclease fragments to high specific activity. *Anal. Biochem.*, **132**, 6.

FIRNHABER, C., GERBER, M. TOOLEY, K. & SCOGGIN, C. (1986). DNA contamination in commercial restriction endonucleases. *Am. J. Hum. Genet.*, **39**, 145.

HENK, J.M. & LANGDON, J.D. (1985). *Malignant tumours of the oral cavity.* Arnold.

HEILMAN, C.A., LAW, M.F., ISRAEL, M.A. & HOWLEY, P.M. (1980). Cloning of human papilloma virus genomic DNA's and analysis of homologous polynucleotide sequences. *J. Virol.*, **36**, 395.

JARRETT, W.F.H., MCNEIL, P.E., GRIMSHAW, W.T.R., SELMAN, I.E. & MCINTYRE, W.I.M. (1978). High incidence area of cattle cancer with a possible interaction between an environmental carcinogen and a papilloma virus. *Nature*, **274**, 215.

LEHN, H., KRIEG, P. & SAUER, G. (1985). Papillomavirus genomes in human cervical tumours: Analysis of their transcriptional activity. *Proc. Nat. Acad. Sci. USA*, **82**, 5540.

LONING, T.H., IKENBERG, H., BECKER, J., GISSMAN, L., HOEPFNER, I. & ZUR HAUSEN, H. (1985). Analysis of oral papillomas, leukoplakias and invasive carcinomas for human papillomavirus type related DNA. *J. Invest. Dermatol.*, **88**, 417.

MAITLAND, N.J., KINROSS, J.H., BUSUTTIL, A., LUDGATE, S.M., SMART, G.E. & JONES, K.W. (1981). The detection of DNA tumour virus-specific RNA sequences in abnormal human cervical biopsies by *in situ* hybridization. *J. Gen. Virol.*, **55**, 123.

IKAWA, H., KAWANA, T. & YOSHIKE, K. (1986). Cloning of monomeric human papillomavirus type 16 DNA integrated within cell DNA from a cervical carcinoma. *J. Virol.*, **58**, 979.

MCCANCE, D.J., KALACHE, A., ASHDOWN, K. & 4 others (1986). Human papillomavirus types 16 and 18 in carcinomas of the penis from Brazil. *Int. J. Cancer*, **37**, 55.

MEANWELL, C.A., COX, M.F., BLACKLEDGE, G. & MAITLAND, N.J. (1987). HPV 16 DNA in normal and malignant cervical epithelium: Implications for the aetiology and behaviour of cervical neoplasia. *Lancet*, **i**, 703.

MOAR, M.H., CAMPO, M.S., LAIRD, H. & JARRETT, W.F.H. (1981). Unintegrated viral DNA sequences in hamster tumour induced by bovine papilloma virus. *J. Virol.*, **39**, 945.

NAHMAIS, A.J., NAIB, Z.M. & JOSEY, W.E. (1974). Epidemiological studies relating genital herpetic infection to cervical carcinoma. *Cancer Res.*, **34**, 1111.

PEDEN, K., MOUNTS, P. & HAYWARD, G.S. (1982). Homology between mammalian cell DNA sequences and human herpesvirus genomes detected by a hybridization procedure with a high complexity probe. *Cell*, **31**, 71.

PFISTER, H. (1984). Biology and Biochemistry of Papillomavirus. *B. Rev. Physiol. Biochem. Pharmacol.*, **99**, 112.

PINDBORG, J.J. (1984). Control of oral cancer in developing countries. *Bull. WHO*, **62**, 817.

SCHNEIDER, A., KRAUS, H., SCHUHMANN, R. & GISSMAN, L. (1985). Papilloma infection of the lower genital tract: Detection of viral DNA in gynaecological swabs. *Int. J. Cancer*, **35**, 443.

SCHWARZ, E., FREESE, U.K., GISSMAN, L. & 4 others. Structure and transcription of human papillomavirus sequences in cervical carcinoma cells. *Nature*, **314**, 111.

SCULLY, C., PRIME, S. & MAITLAND, N. (1985). Papillomaviruses: Their possible role in oral disease. *Oral Surg. Oral Med. Oral Pathol.*, **60**, 166.

SHILLITOE, E.J., TARRO, G. & LEHNER, T. (1976). Cell mediated immunity to herpes simplex virus types 1 and 2 in leuloplakia and carcinoma in man. *Oncology*, **33L**, 192.

SMITH, I.W., MAITLAND, N.J., PEUTHERER, J.F. & ROBERTSON, D.H.H. (1981). Restriction enzyme analysis of herpesvirus type 2 DNA. *Lancet*, **ii**, 1424.

YASUMOTO, E., BURKHARDT, A.L., DONINGER, J. & DIPALO, J.A. (1986). Human papillomavirus type 16 DNA induced malignant transformation of N1H 3T3 cells. *J. Virol.*, **57**, 572.

YEE, C., KRISHMAN-HEWLETT, I., BAKER, C.C., SCHLEGEL, R. & HOWLEY, P.R. (1985). Presence and expression of human papillomavirus sequences in human cervical carcinoma cells. *Am. J. Pathol.*, **119**, 361.

ZUR HAUSEN, H. (1977). Human papillomaviruses and their possible role in squamous cell carcinomas. *Curr. Top. Microbiol. Immunol.*, **79**, 1.